# Routine Vaccination Coverage — Worldwide, 2022

**DOI:** 10.15585/mmwr.mm7243a1

**Published:** 2023-10-27

**Authors:** Gurpreet Kaur, M. Carolina Danovaro-Holliday, George Mwinnyaa, Marta Gacic-Dobo, Lauren Francis, Jan Grevendonk, Samir V Sodha, Ciara Sugerman, Aaron Wallace

**Affiliations:** ^1^Epidemic Intelligence Service, CDC; ^2^Department of Immunization, Vaccines and Biologicals, World Health Organization, Geneva, Switzerland; ^3^Division of Data Analytics, Planning and Monitoring, UNICEF, New York, New York; ^4^Global Immunization Division, Center for Global Health, CDC.

**Summary**

**What is already known about this topic?**

The COVID-19 pandemic negatively affected global childhood immunization programs, resulting in lower childhood vaccination coverage.

**What is added by this report?**

From 2021 to 2022, global coverage with the first dose of diphtheria-tetanus-pertussis–containing vaccine increased from 86% to 89%, and with the first dose of measles-containing vaccine from 81% to 84%, but neither returned to 2019 prepandemic coverage levels of 90% and 86%, respectively. Coverage recovery was unevenly distributed across regions and countries and slower among low-income countries.

**What are the implications for public health practice?**

Strategies to provide catch-up vaccination throughout childhood have the potential to address heightened risks for vaccine-preventable disease outbreaks resulting from years of low vaccination coverage.

## Abstract

In 2020, the World Health Assembly endorsed the Immunization Agenda 2030 (IA2030), the 2021–2030 global strategy that envisions a world where everyone, everywhere, at every age, fully benefits from vaccines. This report reviews trends in World Health Organization and UNICEF immunization coverage estimates at global, regional, and national levels through 2022 and documents progress toward improving coverage with respect to the IA2030 strategy, which aims to reduce the number of children who have not received the first dose of a diphtheria-tetanus-pertussis–containing vaccine (DTPcv1) worldwide by 50% and to increase coverage with 3 diphtheria-tetanus-pertussis–containing vaccine doses (DTPcv3) to 90%. Worldwide, coverage ≥1 dose of DTPcv1 increased from 86% in 2021 to 89% in 2022 but remained below the 90% coverage achieved in 2019. Estimated DTPcv3 coverage increased from 81% in 2021 to 84% in 2022 but also remained below the 2019 coverage of 86%. Worldwide in 2022, 14.3 million children were not vaccinated with DTPcv1, a 21% decrease from 18.1 million in 2021, but an 11% increase from 12.9 million in 2019. Most children (84%) who did not receive DTPcv1 in 2022 lived in low- and lower-middle–income countries. COVID-19 pandemic–associated immunization recovery occurred in 2022 at the global level, but progress was unevenly distributed, especially among low-income countries. Urgent action is needed to provide incompletely vaccinated children with catch-up vaccinations that were missed during the pandemic, restore national vaccination coverage to prepandemic levels, strengthen immunization programs to build resiliency to withstand future unforeseen public health events, and further improve coverage to protect children from vaccine-preventable diseases.

## Introduction

The Expanded Program on Immunization was established by the World Health Organization (WHO) in 1974 to ensure that every infant in the world received vaccines against diphtheria, tetanus, pertussis, poliomyelitis, measles, and tuberculosis (*1*). Since then, immunization programs have broadened to include many additional vaccines.* In 2020, the World Health Assembly endorsed the Immunization Agenda 2030 (IA2030), the 2021–2030 global strategy that envisions a world where everyone, everywhere, at every age, fully benefits from vaccines. A central target of IA2030 is reducing the number of children who have not received the first dose of a diphtheria-tetanus-pertussis–containing vaccine (DTPcv1) (zero-dose children) by 50% by 2030 (*2*). Initial IA2030 implementation was disrupted by the COVID-19 pandemic, and global vaccination coverage declined to the lowest levels in more than a decade, resulting in a 40% increase in the number of zero-dose children during 2019–2021, with fewer vaccinations administered in 2021 compared with 2020 (*3*). This report updates a previous report, reviews global vaccination coverage trends through 2022, and highlights signs of global but uneven immunization program recovery in 2022 (*4*,*5*).

## Methods

WHO and UNICEF produce Estimates of National Immunization Coverage (WUENIC) at the national, regional, and global level based on review of country-specific data, including administrative and survey-based coverage^†^ (*6*,*7*). This report examines trends in coverage with vaccines received from routine immunization programs through 2022, across all WHO countries, as well as aggregated trends at WHO regional and global levels.^§^ Trends in vaccine coverage are also examined by World Bank economic classification.^¶^ Reviewed vaccines include those typically provided by a national routine immunization program during the first year of life: Bacille Calmette-Guérin (BCG); DTPcv1, a third DTPcv dose (DTPcv3); a hepatitis B birth dose (HepB-BD) and third dose (HepB3); a third dose of *Haemophilus influenzae* type b vaccine (Hib3); a first dose of measles-containing vaccine (MCV1); a third pneumococcal conjugate vaccine dose (PCV3); a third polio vaccine dose (Pol3); rotavirus vaccine last dose (Rota, last); and a first rubella-containing vaccine dose (RCV1). Reviewed vaccines provided beyond the first year of life include the second dose of measles-containing vaccine (MCV2), and first and last doses of HPV vaccine (HPV, first; HPV, last).** Zero-dose children represent those who lack access to or are never reached by immunization services (*2*). Children who receive DTPcv1 but not DTPcv3 are considered incompletely vaccinated.^††^ DTPcv1-to-DTPcv3 and DTPcv1-to-MCV1 dropout rates were calculated as the percentage of children who received DTPcv1 but not DTPcv3 or MCV1, respectively.^§§^ This activity was reviewed by CDC, deemed not research, and was conducted consistent with applicable federal law and CDC policy.^¶¶^

## Results

### Diphtheria-Tetanus-Pertussis–Containing Vaccines

WHO and UNICEF estimates of global DTPcv1 coverage increased from 86% in 2021 to 89% in 2022, but remained below 2019 coverage (90%) (Figure). Similarly, estimated DTPcv3 coverage increased from 81% in 2021 to 84% in 2022, but remained below the 2019 level (86%). During 2021 and 2022, DTPcv1 and DTPcv3 coverage improved in all WHO regions except the African Region (AFR), where DTPcv1 and DTPcv3 coverage stagnated at 80% and 72%, respectively, and remained below 2019 coverage (83% and 77%, respectively). During both 2021 and 2022, in the European Region, DTPcv1 and DTPcv3 coverage remained ≥97% and ≥94%, respectively. The South-East Asia Region experienced the most recovery from 2021 to 2022, with DTPcv1 coverage increasing from 86% to 93%, and DTPcv3 coverage increasing from 82% to 91%. Among the 194 WHO countries, 73 (38%) experienced at least a 5% decline in DTPcv3 coverage from 2019 to 2021; among these 73 countries, only 15 (21%) achieved DTPcv3 coverage in 2022 that equaled or exceeded that in 2019.

**FIGURE:**
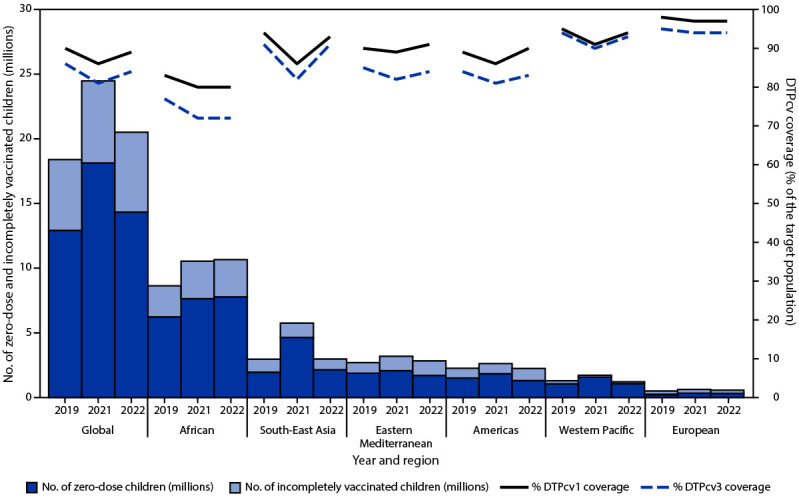
Estimated number of zero-dose and incompletely vaccinated children* and estimated coverage with first and third dose of diphtheria-tetanus-pertussis–containing vaccine, by World Health Organization Region — worldwide, 2019, 2021, and 2022 **Abbreviations:** DTPcv1 = first dose of diphtheria-tetanus-pertussis-containing vaccine; DTPcv3 = third dose of diphtheria-tetanus-pertussis-containing vaccine. * Zero-dose children are surviving children who lack documentation of receipt of any dose of DTPcv by age 12 months (i.e., DTPcv1). Incompletely vaccinated children are those who received at least DTPcv1 but not DTPcv3.

In 2022, the number of zero-dose children (14.3 million) decreased 21%, from 18.1 million in 2021, but was still 11% higher than the 12.9 million in 2019. Only AFR reported an increase in zero-dose children from 2021 to 2022 (2.6%; from 7.6 million to 7.8 million) (Figure) (Table 1). During 2021 and 2022, the number of incompletely vaccinated children (those who had started, but not completed the 3-dose DTPcv series) worldwide remained relatively unchanged (6.3 and 6.2 million, respectively), but higher than the number in 2019 (5.5 million) (Figure). In 2022, most (84%) zero-dose children lived in low- and lower-middle–income countries, and 71% lived in countries eligible for support from Gavi, the Vaccine Alliance (Table 1).

**TABLE 1:** Numbers and global percentages of surviving infants who did not receive the first dose of diphtheria-tetanus-pertussis–containing vaccine (zero-dose children),* by World Health Organization Region, World Bank economic classification, and Gavi, the Vaccine Alliance eligibility — worldwide, 2019, 2021, and 2022

Year/Characteristic	Global	WHO Region^†^						Income classification^§^				Among Gavi-eligible countries^¶^
		AFR	AMR	EMR	EUR	SEAR	WPR	Low	Lower-middle	Upper-middle	High	
**2019**												
No. of countries	**194**	47	35	21	53	11	27	26	54	52	59	57
No. of surviving infants (millions)	**134.3**	37.0	14.0	18.1	10.5	33.3	21.4	23.0	64.6	35.0	12.0	74.3
Global % of surviving infants	**—**	27.5	10.4	13.5	7.8	24.8	15.9	17.1	48.1	26.0	9.0	55.3
No. of zero-dose children (millions)	**12.9**	6.2	1.5	1.9	0.3	2.0	1.1	3.9	6.8	2.2	0.3	9.0
Global % of zero-dose children	**—**	48.2	11.7	14.6	2.0	15.2	8.3	29.8	52.5	16.7	2.3	69.4
**2021**												
No. of countries	**194**	47	35	21	53	11	27	26	54	52	59	57
No. of surviving infants (millions)	**130.5**	38.1	13.6	18.2	10.2	32.8	17.6	24.0	64.5	30.7	11.8	75.2
Global % of surviving infants	**—**	29.2	10.4	14.0	7.8	25.1	13.5	18.4	49.4	23.5	9.1	57.7
No. of zero-dose children (millions)	**18.1**	7.6	1.8	2.1	0.3	4.6	1.6	4.9	9.9	3.1	0.3	12.4
Global % of zero-dose children	**—**	42.1	10.2	11.5	1.8	25.6	8.7	27.2	54.4	17.2	1.7	68.3
**2022**												
No. of countries	**194**	47	35	21	53	11	27	26	54	52	59	57
No. of surviving infants (millions)	**130.6**	38.6	13.6	18.2	10.1	32.7	17.4	23.3	64.5	30.4	11.8	75.7
Global % of surviving infants	**—**	29.5	10.4	14.0	7.7	25.0	13.4	17.8	49.4	23.3	9.0	58.0
No. of zero-dose children (millions)	**14.3**	7.8	1.3	1.7	0.3	2.1	1.1	4.7	7.3	1.9	0.3	10.2
Global % of zero-dose children	**—**	54.3	9.1	12.0	2.2	15.0	7.5	33.1	50.8	13.4	1.9	71.4

**Abbreviations:** AFR = African Region; AMR = Region of the Americas; DTPcv = diphtheria-tetanus-pertussis–containing vaccine; DTPcv1 = first dose of DTPcv; EMR = Eastern Mediterranean Region; EUR = European Region; GNI = gross national income; SEAR = South-East Asia Region; USD = U.S. dollars; WHO = World Health Organization; WPR = Western Pacific Region.

* Zero-dose children are surviving children who lack documentation of receipt of a dose of DTPcv1 by age 12 months. The 2022 WHO and UNICEF estimates of national immunization coverage used the 2022 World Population Prospect from the United Nations Population Division for estimates of national immunization coverage and for calculations of regional and global vaccination coverage figures. Estimates of live births and surviving infants in the 2022 World Population Prospect changed from previous years.

^†^ Included countries are WHO countries.

^§^ Economic classification is based on 2022 GNI per capita, calculated using the World Bank Atlas method in USD (https://datahelpdesk.worldbank.org/knowledgebase/articles/906519-world-bank-country-and-lending-groups). Categorization is based on the World Bank’s economic classification for 2022, in which low-income economies are defined as those with GNI in USD per capita of ≤$1,135; lower middle-income economies, GNI = $1,136–$4,465; upper middle-income, GNI = $4,466–$13,845; and high-income, GNI >$13,845. For all years shown, Cook Islands and Niue are excluded in this classification because of lack of available GNI estimates. For 2021 and 2022, data for Venezuela were also excluded as temporarily unclassified pending release of revised national accounts statistics.

^¶^ Gavi is a public-private global health partnership that aims to increase access to immunization in poor countries. Eligibility is defined as a country’s average 3-year GNI per capita in USD. As GNI increases, a country moves through Gavi’s different eligibility phases until reaching the transition phase in which GNI exceeds the eligibility threshold (https://www.gavi.org/types-support/sustainability/eligibility). Gavi operates on a 5-year strategic period. For Gavi 4.0 (2016–2020), the number of countries decreased to 68 with the same GNI per capita threshold. For Gavi 5.0 (2021–2025), the number of countries remained at 57 but average 3-year GNI per capita threshold was increased to ≤$1,630. For analysis, this report retrospectively looks back at the current 57 Gavi-funded countries to compare across years.

### Measles–Containing Vaccines

From 2021 to 2022, global MCV1 coverage increased from 81% to 83% yet remained below the 2019 coverage level (86%). MCV1 coverage in all regions was lower in 2022 than in 2019, except for the Eastern Mediterranean Region, where it had returned to the 2019 prepandemic level (83%).

Among all 194 WHO countries, 115 (59%) reported lower MCV1 coverage in 2022 than in 2019. Global MCV2 coverage increased from 71% in 2019 to 74% in 2022, principally reflecting the introduction of MCV2 in 11 countries, mostly in AFR, during 2019–2022 (Table 2).

**TABLE 2:** Estimated vaccination coverage, by World Health Organization Region, vaccine, and dose in series — worldwide, 2022

Vaccine	Countries with vaccine in schedule,* no. (%)	Coverage, %						
		Global	WHO Region^†,§,¶^					
			AFR	AMR	EMR	EUR	SEAR	WPR
BCG	155 (80)	**87**	80	87	90	93	91	92
DTPcv1	194 (100)	**89**	80	90	91	97	93	94
DTPcv3	194 (100)	**84**	72	83	84	94	91	93
HepB-BD	103 (53)	**45**	18	65	32	42	58	80
HepB3	190 (98)	**84**	72	83	84	91	91	93
Hib3	193 (99)	**76**	72	83	84	93	91	32
HPV, first**	130 (67)	**21**	33	68	2	37	5	5
HPV, last^††^	130 (67)	**15**	22	52	0	32	3	3
MCV1	194 (100)	**83**	69	84	83	93	92	92
MCV2	188 (97)	**74**	45	76	78	91	85	91
PCV3	157 (81)	**60**	68	78	55	83	58	23
Pol3	194 (100)	**84**	71	82	85	94	91	91
RCV1	173 (89)	**68**	36	84	42	93	92	92
Rota, last^§§^	120 (62)	**51**	51	74	58	31	68	4

**Abbreviations:** AFR = African Region; AMR = Region of the Americas; BCG = Bacille Calmette-Guérin vaccine; DTPcv1 = first dose of diphtheria-tetanus-pertussis–containing vaccine; DTPcv3 = third dose of diphtheria-tetanus-pertussis–containing vaccine; EMR = Eastern Mediterranean Region; EUR = European Region; HepB-BD = birth dose of hepatitis B vaccine; HepB3 = third dose of hepatitis B vaccine; Hib3 = third dose of *Haemophilus influenzae* type b vaccine; HPV, first = first dose of human papillomavirus vaccine; HPV, last = final dose of HPV vaccine; MCV1 = first dose of measles-containing vaccine; MCV2 = second dose of measles-containing vaccine; PCV3 = third dose of pneumococcal conjugate vaccine; Pol3 = third dose of polio vaccine; RCV1 = first dose of rubella-containing vaccine; Rota, last = final dose of rotavirus vaccine series; SEAR = South-East Asia Region; WPR = Western Pacific Region; WHO = World Health Organization.

* Vaccination coverage is reported among the 194 WHO countries. By WHO Region, this includes 47 countries in AFR; 35 countries in AMR; 21 countries in EMR; 53 countries in EUR; 11 countries in SEAR; and 27 countries WPR. Vaccine coverage does not include countries recommending vaccines for special groups only.

^†^ https://www.who.int/about/who-we-are/regional-offices

^§^ https://www.who.int/teams/immunization-vaccines-and-biologicals/policies/who-recommendations-for-routine-immunization---summary-tables

^¶^ Vaccine coverage for all vaccines (except for BCG and HepB-BD) is based on 194 WHO countries (global) or all WHO countries in the specified region. BCG coverage is based on 157 countries that have BCG in the national schedule for all infants. HepB-BD is reported for countries that are able to distinguish vaccine administration within 24 hours of birth. Administrative coverage is the number of vaccine doses administered to those in a specified target group divided by the estimated target population. During vaccination coverage surveys, a representative sample of households are visited, and caregivers of children in a specified target group (e.g., caregivers of children aged 12–23 months) are interviewed. Dates of vaccination are transcribed from the child’s home-based record, from health facility records, or based on caregiver recall. Survey-based vaccination coverage is calculated as the proportion of persons in a target age group who received a vaccine dose.

** Estimates are based on HPV, first dose coverage among females. Number of doses to complete the HPV series depends on the age of the recipient and whether the country has a 1- versus 2-dose HPV immunization policy.

^††^ Estimates are based on HPV, last dose coverage among females. https://www.who.int/teams/immunization-vaccines-and-biologicals/policies/position-papers/human-papillomavirus-(hpv)

^§§^ Number of doses to complete the rotavirus vaccine series varies from 2 to 3, depending on the vaccine product.

### Other Vaccines

Global coverage with the following childhood vaccines increased from 2021 to 2022, but coverage levels in 2022 remained lower than those in 2019: BCG (87%), HepB3 (84%), Pol3 (84%), and RCV1 (68%) remained lower than in 2019 (89%, 86%, 87%, and 69%, respectively). As a result of recent vaccine introductions, coverage with the following vaccines increased from 2019 to 2022: HPV, first (19% to 21%); HPV, last (14% to 15%); PCV3 (51% to 60%); and Rota, last (40% to 51%). Coverage remained relatively unchanged from 2012 to 2022 for HepB-BD (from 44% to 45%) and Hib3 (74% to 76%).

### Vaccination Dropout Rates

In 2022, the DTPcv1-to-DTPcv3 dropout rate (the percentage of children who received DTPcv1 but did not receive DTPcv3) was higher among low-income countries (12%) than among lower-middle–income (5%), upper-middle–income (3%), or high-income (3%) countries. Global DTPcv1-to-MCV1 dropout (the percentage of children who received DTPcv1 but did not receive MCV1) increased from 5% (6.3 million children) in 2019, to 7% (7.6 million) in 2022. In 2022, low-income countries reported the highest DTPcv1-to-MCV1 dropout (17%), substantially higher than that in lower-middle–income (5%), upper-middle–income (3%), and high-income countries (5%) (Supplementary Figure, https://stacks.cdc.gov/view/cdc/134102).

## Discussion

Recovery of global childhood coverage with multiple vaccines occurred from 2021 to 2022; however, recovery was uneven across countries, and some countries have yet to regain 2019 prepandemic coverage levels (*4*). Global distribution of zero-dose and incompletely vaccinated children in 2022 highlights equity issues in immunization coverage and ongoing challenges faced by many low- and lower-middle–income countries. Faster-growing birth cohorts in low- and lower-middle–income countries*** compared with those in upper-middle and high-income countries (*8*) might affect coverage recovery, because some of these countries have vaccinated a similar number of children in 2022 and 2019 in the context of substantially larger 2022 birth cohorts. Although coverage increased from 2021 to 2022 in lower-middle–income countries, where nearly one half of the world’s zero-dose children live, low-income countries experienced higher DTPcv1-to-DTPcv3 dropout rates and little change in the number of zero-dose children, reflecting an uneven recovery.

The COVID-19 pandemic affected immunization programs worldwide and resulted in millions of children missing vaccine doses and substantial increases in the numbers of zero-dose and incompletely vaccinated children. As these children age out of the usual target age range for their country’s routine immunization program, they might experience limited opportunities for catch-up vaccination unless countries adopt catch-up vaccination schedules and strategies for older children. Declines in vaccination coverage among current and older age cohorts can result in immunity gaps and increased risk for outbreaks of vaccine-preventable diseases (*3*). Decreasing vaccinations for measles from 2019 to 2022, especially among low-income countries, contributed to an increase in measles outbreaks in 2022 (*3*). Many countries are implementing catch-up vaccination activities; however, because most national assessments have not typically estimated coverage beyond the usual recommended age range for administration of a given vaccine, it is unclear how effectively pandemic-associated immunity gaps among children older than the recommended age for receipt of a particular vaccine are being reduced through national catch-up vaccination efforts.

To reduce the number of zero-dose children and decrease the number of vaccine-preventable disease outbreaks worldwide (e.g., diphtheria, measles, polio, and yellow fever) will require sustained improvement in immunization coverage and progress toward reaching equity in access across all countries, not only regaining 2019 immunization coverage levels that declined during the pandemic, but also improving immunization coverage beyond 2019 prepandemic levels (*2*). Achieving these goals will require targeted, country-specific strategies, because zero-dose and undervaccinated children tend to live predominantly in low- and lower-middle–income countries and underserved communities; this includes the urban poor and those living in remote rural or conflict-affected settings (*9*). WHO and UNICEF recommend that countries enhance their immunization programs to bolster resiliency against public health events such as the COVID-19 pandemic. Building a resilient program requires actions that include strengthening the health care workforce capacity, ensuring reliable vaccine supply chains, and building community demand and confidence in vaccines. Sustainable program funding and use of immunization data for action will also be needed to identify and reach unvaccinated and undervaccinated children with all recommended and catch-up vaccination opportunities across the lifespan (*3*,*8*).

### Limitations

The findings in this report are subject to at least six limitations. First, for 12 countries (2.2% of the global birth cohort) that did not report 2022 immunization coverage data by July 23, 2023, WUENIC reflects the 2021 estimated coverage (*10*). Second, data quality limitations might have resulted in inaccurate estimates of administrative coverage in some countries. Third, selection and recall bias might affect survey-based estimates of coverage (*7*). Fourth, coverage estimates do not include statistical uncertainty. Fifth, because of COVID-19 pandemic–related disruptions in survey implementation, 2022 estimates are less guided by survey data than are estimates for previous years in this report. Finally, population estimates used to calculate the number of zero-dose and incompletely vaccinated children are subject to inaccuracies.

### Implications for Public Health Practice

The disruptions in daily living and health services during the COVID-19 pandemic set back decades of progress in global immunization activities. Although some recovery was seen in 2022 at the global level, progress was uneven across countries, especially among low- and lower-middle–income countries. Urgent action is needed to provide catch-up vaccination to incompletely vaccinated children, restore national vaccination coverage, and strengthen immunization programs to build the resiliency to withstand future public health events.

## Footnotes

* Additional vaccines recommended by WHO based on region or population risk group. https://cdn.who.int/media/docs/default-source/immunization/immunization_schedules/table_1_feb_2023_english.pdf

† Administrative coverage with a given vaccine is calculated as the number of doses administered in a specified target group divided by the estimated target population. Doses administered during routine immunization visits are counted, but doses administered during supplementary immunization activities (mass campaigns) usually are not. Survey-based vaccination coverage is calculated as the proportion of persons in a target age group who had received a vaccine dose. During surveys, a representative sample of households is visited, and caregivers of children in a specified target age group (e.g., 12–23 months) are interviewed. Vaccination dates are transcribed from the child’s home-based record or health facility records, and if documented evidence is unavailable, recorded based on caregiver recall.

§ Years 2021 and 2022 are reported for the pandemic period: 2021, because immunization coverage in 2021 was worse than 2020; and 2022, as the most recent update.

¶ Economic classification is based on gross national income (GNI) per capita, calculated using the World Bank Atlas method (https://datahelpdesk.worldbank.org/knowledgebase/articles/906519-world-bank-country-and-lending-groups). Low-income economies are defined as those with GNI in U.S. dollars per capita in 2022 of <$13,845; lower-middle–income, $1,136–$4,465; upper-middle–income, $4,466–$13,845; and high-income, >$13,845. For all years shown, Cook Islands and Niue are excluded in this classification because of lack of available GNI estimates. For 2021 and 2022, data for Venezuela were also excluded as temporarily unclassified pending release of revised national accounts statistics.

** Vaccines provided during the first year of life versus after the first year of life are based on WHO routine immunization schedule (https://cdn.who.int/media/docs/default-source/immunization/immunization_schedules/table_1_feb_2023_english.pdf?sfvrsn). The age at which a specific vaccine is delivered might vary among different country routine immunization schedules based on its associated country-specific disease incidence.

†† Incompletely vaccinated children might also be referred to as undervaccinated children.

§§ DTPcv1-to-DTPcv3 dropout = ([number of children vaccinated with DTPcv1] – [number of children vaccinated with DTPcv3]) / (number of children vaccinated with DTPcv1) x 100%. DTPcv1-to-MCV1 dropout = ([number of children vaccinated with DTPcv1] – [number of children vaccinated with MCV1]) / (number of children vaccinated with DTPcv1) x 100%.

¶¶ 45 C.F.R. part 46.102(l)(2), 21 C.F.R. part 56; 42 U.S.C. Sect. 241(d); 5 U.S.C. Sect. 552a; 44 U.S.C. Sect. 3501 et seq.

*** Annual population growth rate (%) is highest in West and Central Africa (2.7%), sub-Saharan Africa (2.6%), and Eastern and Southern Africa (2.5%). Global projected population growth rate during 2020–2030 is also highest among these African countries (https://www.unicef.org/media/108161/file/SOWC-2023-full-report-English.pdf). Among the 26 low-income countries and 54 lower-middle–income countries, 20 (77%) and 19 (35%) are in AFR, respectively.
